# Immunomodulatory Effects of Different Lactic Acid Bacteria on Allergic Response and Its Relationship with *In Vitro* Properties

**DOI:** 10.1371/journal.pone.0164697

**Published:** 2016-10-20

**Authors:** Chunqing Ai, Na Ma, Qiuxiang Zhang, Gang Wang, Xiaoming Liu, Fengwei Tian, Pei Chen, Wei Chen

**Affiliations:** 1 National Engineering Research Center of Seafood, School of Food Science and Technology, Dalian Polytechnic University, Dalian 116034, P. R. China; 2 State Key Laboratory of Food Science and Technology, School of Food Science and Technology, Jiangnan University, Wuxi 214122, P. R. China; 3 Shaanxi University of Technology, School of Biological Science and Engineering, Hanzhong 723001, P. R. China; 4 Shanxi Radio & TV University, Xi'an 710119, P. R. China; Kyoto Daigaku, JAPAN

## Abstract

Some studies reported that probiotic could relieve allergy-induced damage to the host, but how to get a useful probiotic is still a challenge. In this study, the protective effects of three lactic acid bacteria (La, Lp and Lc) were evaluated in a mouse model, and its relationship with the *in vitro* properties was analyzed. The *in vitro* results indicated that La with the capacity to inhibit IL-4 production could have a better anti-allergy effect *in vivo* than two others. However, the animal trials showed that all LAB strains could alleviate allergen-induced airway inflammation. Among them, LAB strain Lp had a better effect in inhibiting allergic response through a modulation of Th1/Th2 balance and an increase of regulatory T cells. This difference could be explained by that different LAB strains have a strain-specific effect on gut microbiota closely associated with host immune responses. Finally, this study did not only obtain an effective anti-allergy probiotic strain via animal study, but also indicate that probiotic-induced effect on intestinal microbiota should be considered as an important screening index, apart from its inherent characteristics.

## Introduction

All around the world, allergic diseases have become an important public health issue that affects more than 25% of the world’s population in both developed and developing countries [1]. In recent years, the prevalence of type I allergy is increasing annually throughout the world, seriously affecting people’s quality of life. Such an increase could be attributed to a lack of microbial stimulation of the infant gut immune system [2]. Almost immediately after a human being is born, a new microbial ecosystem is shaped in person’s gastrointestinal tract, which serves numerous important functions for host, including maturation of host immune system, nutrient processing and protection against infectious agent. As already reported, human microbiota is composed of approximately 10^14^ microbes, which is ten times more microbial cells than human cells. Among numerous intestinal microbes, lactic acid bacteria (LAB) as a key component first appear in the infant gut, and its number and species composition are closely associated with the healthy state [3, 4].

LAB strain has been widely used in the fermentation and storage of some foods for thousands of years, such as milk, meat and vegetables. With in-depth studies on LAB, it is not only considered as fermentation starters, but major species qualify as probiotic defined as living microorganism that, on ingestion in adequate amounts, exert health effect beyond inherent basic nutrition [5]. Schiavi et al. [6] demonstrated that a probiotic mixture could effectively suppress established Th2 responses and systemic anaphylaxis in a mouse model of food allergy. Some clinical trials also indicated that probiotic administration early in life could be effective in reducing the risk of IgE-associated allergic diseases in infants [7, 8]. Besides that, specific LAB strain was also reported to exert health-promoting effect on some other aspects, such as inflammatory bowel diseases, infectious diseases and metabolic diseases. Based on its immunomodulatory properties, probiotic therapy is considered to be an effective supplement or potential candidate for the prevention and treatment of some immune-related disorders.

However, not all LAB strains were proven to be effective in modulating the Th1/Th2 immune balance to inhibit allergen-induced allergic responses [9]. It has been accepted that the immunomodulatory activities induced by LAB strains are different among species, and even between different strains of same species [10]. To obtain specific strain with anti-allergy characteristics, an *in vitro* model is always used as a rapid screening method to identify potential candidate from numerous LAB strains, and then the protective effect of selected strain is further evaluated in an animal model [11]. Typical characteristics of type I allergy is a shift in the Th1/Th2 balance towards to Th2-dominated response characterized by an increase in the levels of Th2 cytokines IL-4 and IL-5 [12]. Therefore, some studies suggested that LAB strains capable of inhibiting IL-4 expression *in vitro* could perform an anti-allergy effect *in vivo* [13, 14]. So far, there are only a few studies comparing potential probiotic strains *in vitro* and *in vivo*, but the accuracy of this screening model is not given enough attention.

In this study, the immunomodulatory properties of three LAB strains, isolated from traditional fermented foods Paocai, were first analyzed through an *in vitro* co-culture with spleen lymphocytes. Subsequently, house dust mite (HDM) allergen Der p2 was used to construct a mouse allergy model, and the anti-allergy activities induced by different LAB strains were systemically investigated by measuring the levels of cytokines, antibodies, regulatory T cells, inflammatory responses in lung tissues and species composition of intestinal microbiota. By analyzing the *in vitro* and *in vivo* studies, the relationship between them were systemically discussed, which would be helpful in the screening of probiotic strain with specific characteristics.

## Materials and Methods

### Bacterial strains

Three different strains *Lactobacillus plantarum* CCFM47 (Lp), *Lactobacillus acidophilus* CCFM137 (La) and *Lactobacillus casei* Lc2w (Lc) were isolated from traditional fermented foods Paocai, and have proven to have the immunomodulatory properties [15]. For the actual experiment, all bacteria were anaerobically grown in de Man Rogosa and Sharpe (MRS) broth to stationary phase at 37°C, pelleted by centrifugation and resuspended in 0.1M PBS buffer (pH 7.4). The bacterial density in suspension was determined by the dilution plate counting method.

### *In vitro* co-culture of spleen cells with LAB strains

Fresh spleen cells isolated from normal BALB/c mice were co-cultured with single LAB strain *in vitro* as mentioned previously with a little modification [16]. The isolated spleen cells were cultured in RPMI 1640 complete medium, and cell density was adjusted to 1×10^6^ cells/mL. Cell cultures were performed by mixing 200 μL of single cell suspension and 20 μL of bacterial suspension (1×10^7^ or 1×10^8^ cfu/mL) in 96-well plates (NUNC, USA) at 37°C in 5% CO_2_, and all experiments were set up in triplicate. After incubation for 72h, the supernatants were collected and stored at -80°C until further analysis. Concentrations of IL-4, IL-10, IL-12 and IFN-γ in the supernatants were measured by ELISA (Rapidbio Lab, China).

### Protocol of animal experiment

**Fig 1:**
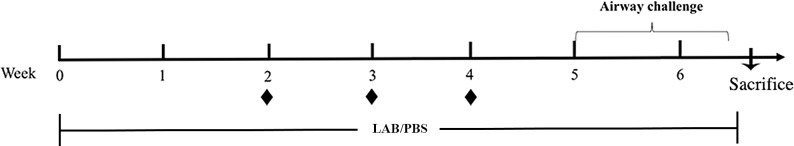
Protocol of mice experiment. Mice were orally administrated with 200 μL/day of bacterial suspension or PBS until mice were sacrificed. At weeks 2, 3 and 4, mice underwent intraperitoneal injection with a mixture of Der p2/Alum or PBS (◆) at weekly intervals. To induce allergic airway inflammation, all mice were inhalation challenged with HDM allergen after the last vaccination.

Thirty-five female BALB/c mice (SPF, 4 weeks) were purchased from Shanghai SLAC Laboratory Animals Co., Ltd., and kept at the Animal Center of Jiangnan University (JN N. 20130327–0605 (26)). The food and sterile water were given *ad libitum*. All mice were randomly divided into five groups, and housed in the standard cages for one week prior to the actual experiment. The mice model was established as previous description by Rigaux et al. [17] with a slight modification. Briefly, three LAB-treated groups were orally administrated with 2×10^9^ cfu/day of La, Lc and Lp strain until mice were sacrificed, and other two groups were fed with sterile PBS. At weeks 2, 3 and 4, all mice except for the control group were intraperitoneally sensitized with 10 μg of Der p2 formulated with 2 mg of Alum at weekly intervals. Seven days after the last vaccination, mice were challenged for 10 days by exposure to aerosolized HDM allergen over a 45min period (Fig 1).

### Ethics statement

All mice experiment was approved by the Animal Ethics Committee of Jiangnan University (JN No. 20121102–0120[25]). The protocol was carried out in strict accordance with the European Community guidelines (Directive 2010/63/EU) for the care and use of experimental mice. During the animal experiments, all mice were monitored everyday and no mice exerted severely allergic symptoms prior to the experimental endpoint. Mice were sacrificed by cervical dislocation with sodium pentobarbital anesthesia, and all efforts were made to minimize suffering.

### Measurement of antibodies and cytokines in serum

The levels of Der p2-specific IgE, IgG1 and IgG2a in serum were analyzed as described by Lee et al. [18]. In addition, the concentrations of total IgE antibody, IL-4 and IFN-γ in serum were measured by ELISA in accordance with the manufacturer’s instructions (Rapidbio Lab, China).

### Analysis of inflammatory responses in the lung tissues

Analysis of inflammatory cytokine IL-4 in bronchoalveolar lavage

Briefly, bronchoalveolar lavage was performed by using 0.8 mL HBSS buffer instilled bilaterally with a syringe. The bronchoalveolar lavage fluid (BALF) was collected three times by gentle aspiration and centrifuged. The level of IL-4 in BALF was evaluated by ELISA (Rapidbio Lab, China).

#### RT-PCR analysis of Foxp3

The mRNA expression level of Foxp3 in lung tissues was analyzed by RT-PCR as previous description [19]. Briefly, total RNA was isolated from liquid nitrogen-frozen tissues with Trizol^®^ reagent, and reverse transcription into cDNA was performed by the QuantScript RT Kit (TIANGEN, China). The level of Foxp3 mRNA was determined using the SYBR Green Kit (TIANGEN, China) with the primers (F-AGAGTTCTTCCACAACATGGACTACTT; R-GATGGCCCATCGGATAAGG). The housekeep gene β-actin (F-TGAGAGGGAAATCGTGCGTGAC; R-GCTCGTT- GCCAATAGTGATGACC) was used as a reference gene. Relative quantification of Foxp3 was calculated by the comparative CT method [20].

#### Histological analysis of lung tissues

The lung tissues isolated from different mice were fixed with 10% neutral phosphate-buffered formalin overnight, and embedded in paraffin. After that, sections were prepared and stained with hematoxylin/eosin for histological evaluation by light microscopy (Leica, Germany).

### Measurement of regulatory T cells

After mice were sacrificed, spleen and mesenteric lymph nodes (MLN) were separately isolated from mice, and turned into single cell suspension by mechanical disruption. After that, cell density was adjusted to 5×10^6^ cells/mL and stained with extracellular CD4 and CD25 antibodies and intracellular Foxp3 antibody (eBioscience, USA) in accordance with the manufacturer’s instructions. For Foxp3 staining, cells were first stained with two surface markers, and then fixed, permeabilized and stained for Foxp3. At last, the staining cells were detected and analyzed by FACS Caliber (BD Bioscience, USA).

### PCR-DGGE analysis of microbiota community composition

#### DNA extraction and PCR amplification

At the end of animal experiment, fecal samples were separately collected from different group and stored at -80°C. DNA was extracted by using Fast DNA Spin Kit for Soil (MP Biomedicals) following the manufacturer’s instruction. The V3 region of 16S rRNA was amplified using the primers (F-CGCCCGCCGCGCGCGGCGGG- CGGGGCGGGGGCACGGGGGGACTCCTACGGGAGGCAGCAG; R-ATTACCG- CGGCTGCTGG) by hot-start touchdown PCR [21]. An aliquot of PCR product was analyzed in 1% agarose gel to confirm the size of DNA fragment.

#### DGGE analysis

The DGGE analysis was carried out using a DCode™ Universal Detection system instrument as previously mentioned [22]. The acrylamide concentration used in the gel was 8%, and the denaturing gradient was from 30% to 65%. Electrophoresis was performed in 1 × TAE buffer at a voltage of 70 V for 16h. After that, gel was stained with Gel Red (Biotium, USA), and digitised in UV light with the Gel Doc™ 2000 Documentation System (Bio-Rad, USA).

#### DNA Sequencing of DGGE bands

Nucleotide sequences of DNA fragments recovered from the bands in DGGE gel were determined by the following method. The gel strip of a band was excised with a razor blade, and retrieved from the acrylamide block in 50 μL TE buffer at 4°C overnight. The DNA was re-amplified with no GC-clamp primers, and cloned by the TA cloning method in pUC19 vector with TA cloning kit (TaKaRa, China). The recombinant plasmids were transformed into *E*. *coli* Top10 in accordance with the manufacturer’s instruction. Finally, the constructed plasmids were extracted from randomly picked clonies with the UNlQ-200 Column Plasmid Medi-Preps Kit (Sangon, China) for subsequent sequence analysis.

### Statistical analysis

The results were expressed as means ± SD, and statistical analysis was performed using SPSS for windows software. Statistical differences between groups were determined by one-way analysis of variance (ANOVA) following Duncan’s test, and p < 0.05 was considered to be statistically significant.

## Results and Discussion

### Immunomodulatory properties of LAB strains *in vitro*

**Fig 2:**
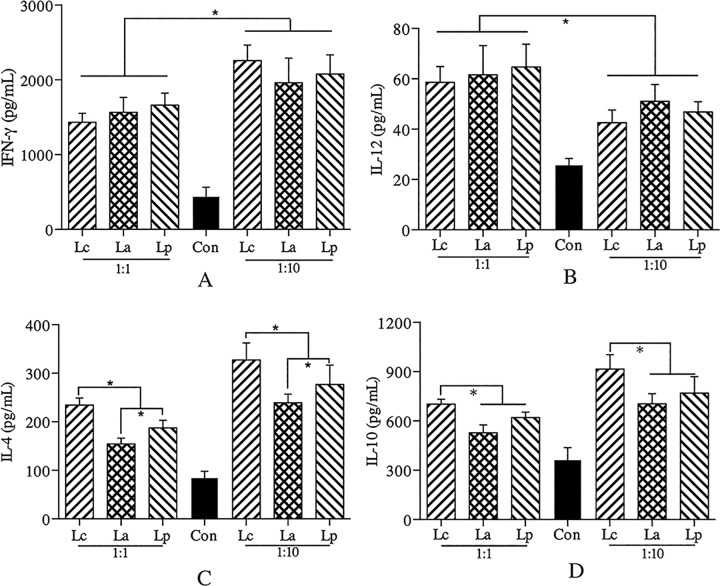
Immunomodulatory properties of different LAB strains in vitro. The levels (pg/mL) of IFN-γ (A), IL-12 (B), IL-4 (C) and IL-10 (D) were measured by ELISA.

There is a growing body of evidence indicating that specific probiotic could promote anti-allergic processes through an induction of Th1 immunity which plays an essential role in the suppression of Th2-induced allergy [23], but how to get an effective strain is still a challenge. A few studies suggested that specific LAB strain with the capacity to inhibit IL-4 production *in vitro* could be a potential anti-allergy strain *in vivo* [10]. All three LAB strains used could stimulate the expression of cytokines, and their immunomodulatory properties could be affected by the ratio of cell/bacteria (Fig 2). Unlike IL-4 and IL-10, there was no significant difference between the LAB strains in the levels of IL-12 and IFN-γ, whatever the cell/bacteria ratio. In contrast to Lc, both La and Lp could induce less IL-4 and IL-10 secretion in the cell cultures, especially La, which was considered to exert better inhibitory effect on allergic responses *in vivo*. To further evaluate the accuracy of this screening model, the protective effects of three LAB strains were systemically analyzed in an established allergy mice model.

### Analysis of serum antibodies

**Fig 3:**
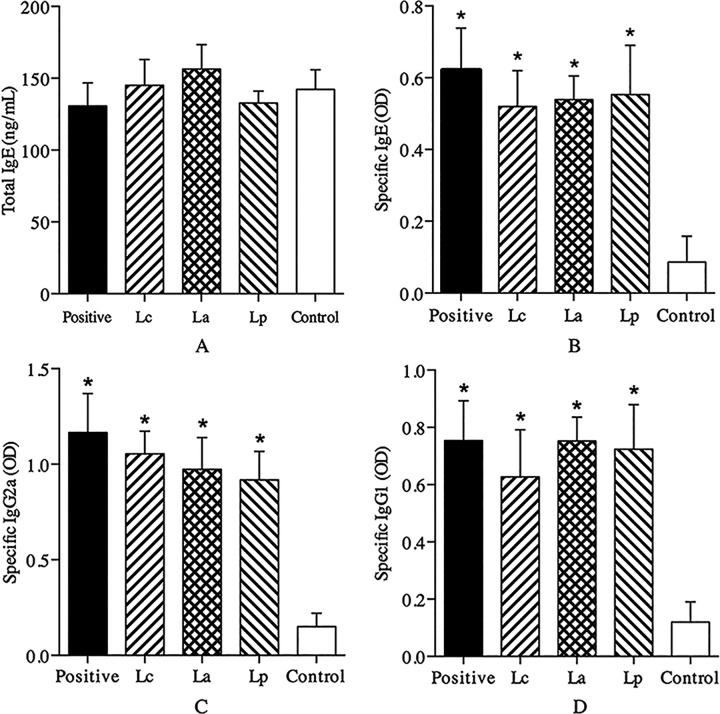
Effects of different LAB strains on antibodies in serum. The levels of total IgE (A) and Der p2-specific IgE (B), IgG2a (C) and IgG1 (D) in serum were respectively measured as ng/mL or optical density (OD) by ELISA. ^*^P < 0.05 vs control group.

Generally, allergen-specific IgE antibody is considered as a surrogate marker for the clinical diagnosis of type I allergic diseases [24]. In this study, Der p2-sensitized mice exerted significant Th2-biased allergic responses characterized by an increased specific IgE response (Fig 3B). Surprisingly, oral treatment with any LAB strains had no remarkable effects on the levels of specific IgE and IgG antibodies (Fig 3B, 3C and 3D). These results differed from previous studies [6, 25] that specific LAB strain can stimulate the IgG2a/IgG1 production to inhibit IgE-mediated allergic response. However, some other studies demonstrated that specific LAB strain could relieve allergen-induced allergic responses independent of IgG antibodies [26]. These conflictive results indicated the immunomodulatory mechanisms of specific LAB strain in anti-allergic processes are complex and strain-dependent.

### Measurement of inflammatory cytokines in serum

**Fig 4:**
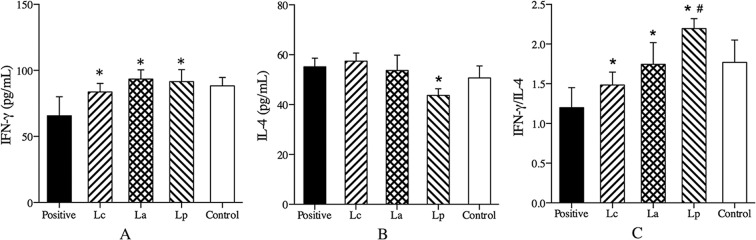
Effects of different LAB strains on cytokines production in serum. The levels of IFN-γ (A), IL-4 (B) and the ratio of IFN-γ/IL-4 (C) were measured by ELISA. ^*^P < 0.05 vs positive group, ^#^P < 0.05 vs La group.

Allergen-reactive Th2 cells and pro-inflammatory cytokines have been suggested to play an important role in the induction and maintain of the inflammatory cascade in allergic disorders [27]. Clinical studies showed that the ratio of Th1/Th2 cytokines (IFN-γ/IL-4) was lower in allergic patients in contrast to those of normal people [28]. Compared with the positive group, oral treatment with any LAB strains could significantly stimulate the production of Th1-associated cytokine IFN-γ in the Der p2-sensitized mice, and no significant difference was found between them (Fig 4A). However, only strain Lp could effectively inhibit Th2 cytokine IL-4 production, and raise the ratio of IFN-γ/IL-4 (Fig 4B and 4C). These results were consistent with that specific LAB strain could relieve Th2-mediated allergic symptom, probably through a modulating effect on the Th1/Th2 immune balance [29, 30].

### Analysis of inflammatory responses in the lung tissues

**Fig 5:**
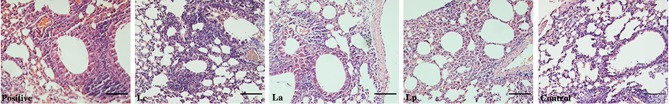
Histological analysis of the lung tissues from positive, Lc, La, Lp, and control group. The bar length was 100 μm and the magnification times was 200.

**Fig 6:**
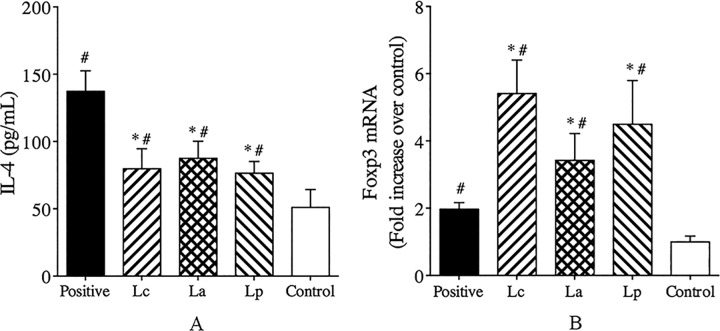
Analysis of inflammatory responses after inhalation challenge. The level of IL-4 (A) in the BALF was measured by ELISA. The mRNA level of Foxp3 (B) in the lung tissues was analyzed by RT-PCR. ^#^P < 0.05 vs control group and ^*^P < 0.05 vs positive group.

HDM allergen-caused allergic asthma is a typical airway inflammatory disease, and it has been recognized that a variety of inflammatory cells play a key role in this process [31]. Histological analysis revealed that repeated inhalation of allergen could significantly induce an accumulation of inflammatory cells in the lung tissues of Der p2-sensitizided mice (Fig 5). By contrast, oral treatment with any LAB strains could lead to a decrease in the inflammatory cells infiltration (Fig 5), which was closely associated with a reduction in IL-4 and an increase in the level of Foxp3 mRNA in lung tissues (Fig 6). Although there was no significant difference in the cellular infiltration and tissue changes (S1 Table), both Lc and Lp could induce a relatively high expression of Foxp3 mRNA and inhibit IL-4 production relative to La-treated mice. Regulatory T cells (Tregs) with Foxp3 as an indicative marker have been reported to modulate Th1/Th2 cytokines profile and cellular infiltration in the inflamed tissues [32, 33]. Feleszko et al. [34] demonstrated that probiotic-induced suppression of allergic airway inflammation was closely associated with an increase of Tregs in the peribronchial lymph nodes. It indicated that probiotic could alleviate allergen-induced inflammatory responses by modulating the profile of Th1/Th2 cytokines in Tregs-dependent mechanism.

### Induction of Tregs in the spleen and MLN

**Fig 7:**
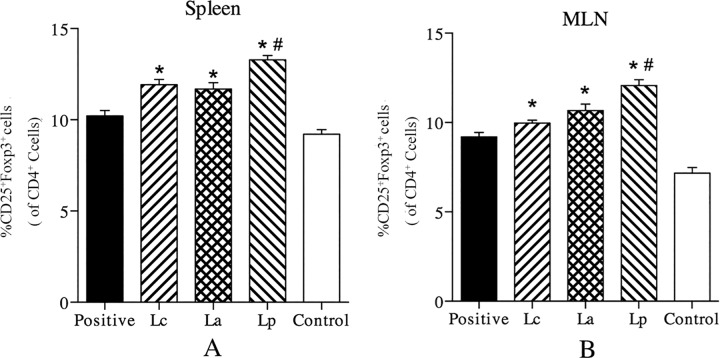
Effects of different LAB strains on the levels of CD4^+^CD25^+^Foxp3^+^ Tregs in the spleen (A) and MLN (B) of Der p2-sensitized mice. ^*^P < 0.05 vs positive group and ^#^P < 0.05 vs La group.

Recent studies have proven that Tregs plays an important role in maintaining peripheral tolerance to environmental harmless antigen, and suppressing an excessive immune responses deleterious to the host [35]. To investigate the effects of LAB strains on Tregs, the levels of CD4^+^CD25^+^Foxp3^+^ cells in the spleen and MLN were analyzed by FACS. The results showed that three LAB strains could increase the proportion of Tregs in the MLN and spleen of Der p2-sensitized mice, especially Lp (Fig 7). Such an increase is believed to play a critical role in the intervention of early development of allergic diseases, which can be explained by that Tregs can inhibit the proliferation and cytokines secretion of effector T cells [36]. Moreover, a population of Tregs can create a milieu which would be helpful in promoting the outgrowth of new Tregs with antigen specificities and distinct from original Tregs [37]. Based on the migration characteristics of Tregs *in vivo* [38], probiotic-induced increase of Foxp3 mRNA in the lung tissues might be associated with an up-regulation of Tregs in the MLN and spleen.

### PCR-DGGE analysis of intestinal microbiota

**Fig 8:**
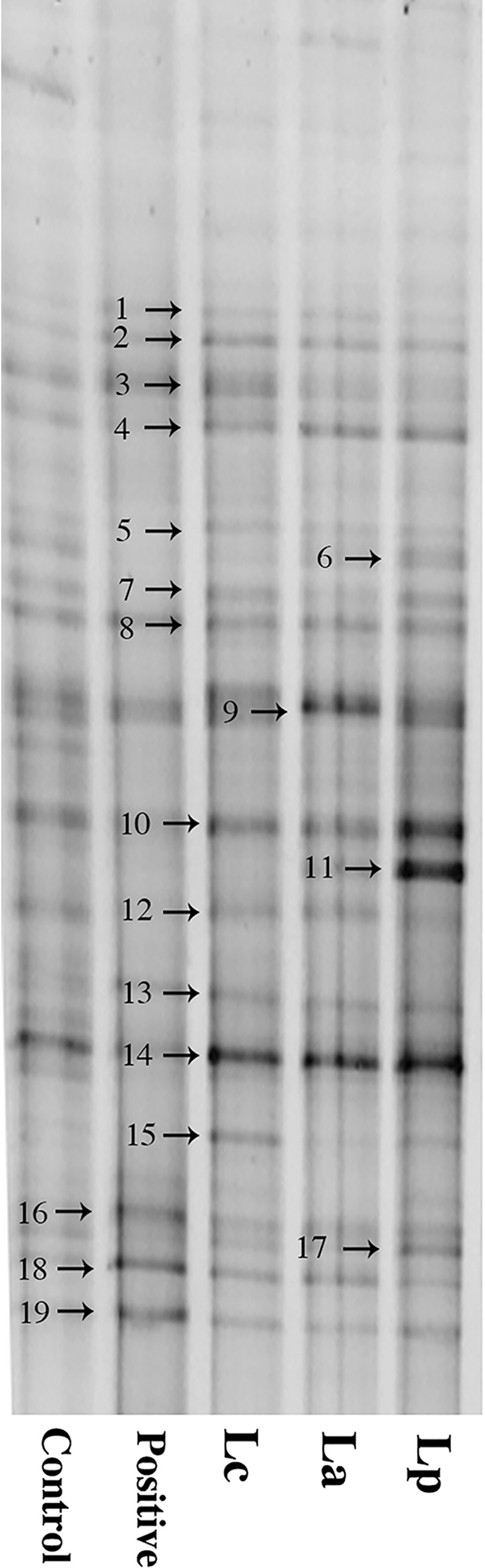
PCR-DGGE analysis of fecal bacteria species composition.

So far, the precise mechanisms by which probiotic regulate the host immune system have still not been fully elucidated, but a growing body of evidence suggested that it could be closely associated with the direct or indirect impact on colonizing microbiota of gut [39]. To determine the effects of different LAB strains on intestinal microbiota, fecal bacteria species composition was analyzed by the PCR-DGGE method. The result revealed that oral application of any LAB strains seemed to have no significant effect on the overall microbiota structure, but the amount of specific species exerted notable changes, especially Lp treatment group (Fig 8). The DNA sequencing of isolated bands indicated that some members of the phyla *Firmicutes* and *Bacteroidetes* were significantly increased in Lp-treated mice compared with the positive group (Table 1). Recent studies have demonstrated that *Firmicutes* and *Bacteroidetes* are two dominant bacterial species in the intestinal tract, which play an important role in the development and maintenance of host immune system [40]. Wang et al. [41] demonstrated that probiotic-mediated attenuation of metabolic syndromes was closely associated with strain-specific impact on the phenotypes of gut microbiota. Therefore, apart from its inherent immunological properties, LAB strain-specific modulation on the microbiota should be considered as a key channel that could affect its *in vivo* action effect.

**Table 1:** Results of blast analysis on DNA sequence from the DGGE bands.

Band	Closest relative	Similary	Phyla
1	*Rikenella microfusus*	94%	*Bacteroidetes*
2	*Bifidobacterium bohemicum*	87%	*Actinobacteria*
3	*Helicobacter pullorum*	100%	*Proteobacteria*
**4**	***Butyrate-producing bacterium***	**99%**	***Firmicutes***
5	*Clostridiales bacterium*	99%	*Firmicutes*
6	*Lachnospiraceae bacterium*	99%	*Firmicutes*
7	*Oscillospiraceae bacterium*	99%	*Firmicutes*
8	*Barnesiella intestinihominis*	90%	*Bacteroidetes*
9	*Clostridiales bacterium*	94%	*Firmicutes*
**10**	***Clostridium polysaccharolyticum***	**99%**	***Firmicutes***
**11**	***Lachnospiraceae bacterium***	**98%**	***Firmicutes***
12	*Butyrivibrio proteoclasticus*	92%	*Firmicutes*
13	*Porphyromonadaceae bacterium*	95%	*Bacteroidetes*
**14**	***Barnesiella intestinihominis******c***	**90%**	***Bacteroidetes***
15	*Johnsonella* *bacterium*	96%	*Firmicutes*
16	*Parabacteroides distasonis*	88%	*Bacteroidetes*
**17**	***Barnesiella intestinihominis***	**89%**	***Bacteroidetes***
18	*Dysgonomonas capnocytophagoides*	89%	*Bacteroidetes*
19	*Clostridiales bacterium*	98%	*Firmicutes*

## Supporting Information

S1 Table The degree of allergic airway inflammation in different groups. (DOCX)
