# Supplementary material for: Immunomodulatory Effects of Different Lactic Acid Bacteria on Allergic Response and Its Relationship with In Vitro Properties
Source: PLoS One. 2016 Oct 20;11(10):e0164697. doi: 10.1371/journal.pone.0164697 (PMC5072832; doi:10.1371/journal.pone.0164697)
Supplement: S1 Table — (DOCX) [file pone.0164697.s001.docx]

**S1 Table** The degree of allergic airway inflammation in different groups

| Group | Grade |
| --- | --- |
| Positive | 4.4±0.5^a^ |
| Lc | 2.4±0.5^b^ |
| La | 2.0±0.6^b^ |
| Lp | 2.2+0.7^b^ |
| Control | 0.6±0.5^c^ |

Allergic pulmonary inflammation induced by HDM allergen was graded 0-5 based on the degree of inflammatory cells infiltration and changes of lung tissues. The mean values within the same column followed by different superscript letters differ significantly (P < 0.05).
